# Molecular allergology in North America; understanding the delay

**DOI:** 10.1186/2045-7022-4-S2-P34

**Published:** 2014-03-17

**Authors:** Guy Tropper

**Affiliations:** 1 AVANT GARDE Médical, 550 Boul. De Mortagne, Suite 260, Boucherville, J4B 5E4, Canada

## 

Was America sleeping on its laurels or just snubbing molecular allergology / component-resolved diagnosis? The clinical implementation of molecular allergology testing in Europe dates back to the early 2000's. Although the importance of major allergens seems to have been readily recognized by allergy professionals in North America, more exhaustive exploration and clinical use of molecular allergology is still waiting. In this day and age of instant communication and worldwide professional collaboration, it is surprising that such a gap could have developed. A review of factors that may have led to this situation represents an opportunity to identify important dynamics that sometimes hinders the introduction of valuable science and/or technologies to our patient care. In France, the model of physician remuneration may have set up the opportunity for the professional cooperation that developed the science and its integration to government-sponsored healthcare services. In Canada, the lack of expertise and demand for molecular allergology services has hindered the country-specific certification process of laboratory technology otherwise widely recognized. In the US, the FDA is still restricting its endorsement to the "Peanut-You-Know" portion of Thermofisher's line of molecular allergy testing products. All the while and until recently, pioneering US-based research in oral immunotherapy (OIT) neglected basic data concerning peanut allergy molecular profiles at a time when comprehensive protocols were routine clinical practice in some French centers. Whereas http://www.allerdata.com and http://www.allergome.com websites provide a bounty of information about molecular allergology, language issues (French/English, basic science/clinical) may have impeded the dissemination of the science. Realities remote from the objective value of molecular allergology / component-resolved diagnosis may explain the gap in clinical implementation between Europe and North America. If the latter is to assert a dominant role in the field of allergy, its professionals will have to embrace change and provide leadership in molecular allergology. As for sublingual immunotherapy, inaction on the part of allergy professionals will leave to commercial and industrial interests the definition of the business model and ultimately the patients' health care options.

